# Potential of Prebiotic D-Tagatose for Prevention of Oral Disease

**DOI:** 10.3389/fcimb.2021.767944

**Published:** 2021-11-05

**Authors:** Shota Mayumi, Masae Kuboniwa, Akito Sakanaka, Ei Hashino, Asuka Ishikawa, Yura Ijima, Atsuo Amano

**Affiliations:** Department of Preventive Dentistry, Osaka University Graduate School of Dentistry, Suita, Japan

**Keywords:** d-tagatose, biofilm, transcriptomics, metabolomics (OMICS), *Streptococcus mutans* (*S. mutans*), *Streptococcus gordonii*, *Streptococcus oralis*

## Abstract

Recent studies have shown phenotypic and metabolic heterogeneity in related species including *Streptococcus oralis*, a typical oral commensal bacterium, *Streptococcus mutans*, a cariogenic bacterium, and *Streptococcus gordonii*, which functions as an accessory pathogen in periodontopathic biofilm. In this study, metabolites characteristically contained in the saliva of individuals with good oral hygiene were determined, after which the effects of an identified prebiotic candidate, D-tagatose, on phenotype, gene expression, and metabolic profiles of those three key bacterial species were investigated. Examinations of the saliva metabolome of 18 systemically healthy volunteers identified salivary D-tagatose as associated with lower dental biofilm abundance in the oral cavity (Spearman’s correlation coefficient; *r* = -0.603, *p* = 0.008), then the effects of D-tagatose on oral streptococci were analyzed *in vitro*. In chemically defined medium (CDM) containing D-tagatose as the sole carbohydrate source, *S. mutans* and *S. gordonii* each showed negligible biofilm formation, whereas significant biofilms were formed in cultures of *S. oralis*. Furthermore, even in the presence of glucose, *S. mutans* and *S. gordonii* showed growth suppression and decreases in the final viable cell count in a D-tagatose concentration-dependent manner. In contrast, no inhibitory effects of D-tagatose on the growth of *S. oralis* were observed. To investigate species-specific inhibition by D-tagatose, the metabolomic profiles of D-tagatose-treated *S. mutans*, *S. gordonii*, and *S. oralis* cells were examined. The intracellular amounts of pyruvate-derived amino acids in *S. mutans* and *S. gordonii*, but not in *S. oralis*, such as branched-chain amino acids and alanine, tended to decrease in the presence of D-tagatose. This phenomenon indicates that D-tagatose inhibits growth of those bacteria by affecting glycolysis and its downstream metabolism. In conclusion, the present study provides evidence that D-tagatose is abundant in saliva of individuals with good oral health. Additionally, experimental results demonstrated that D-tagatose selectively inhibits growth of the oral pathogens *S. mutans* and *S. gordonii*. In contrast, the oral commensal *S. oralis* seemed to be negligibly affected, thus highlighting the potential of administration of D-tagatose as an oral prebiotic for its ability to manipulate the metabolism of those targeted oral streptococci.

## Introduction

Dental caries and periodontal disease are prevalent polymicrobial infections found in the human oral cavity, which often lead to tooth loss and have effects on general health (Lamont and Hajishengallis, 2015; Johansson et al., 2016; Bernabe et al., 2020). Such infections develop when dental biofilm shifts from a symbiotic to dysbiotic state (Lamont et al., 2018) and disruption of biofilm showing symbiosis has been found to be associated with its maturation over time (Mira et al., 2017). During periodontal pathogenic biofilm formation, early colonizers such as streptococci adhere to salivary pellicles on tooth surfaces, followed by accumulation of intermediate colonizers, including *Fusobacterium nucleatum* and periodontopathic bacteria such as *Porphyromonas gingivalis*, in the biofilm (Kuboniwa and Lamont, 2010). In particular, *Streptococcus gordonii* supports progression of periodontal disease due to its symbiotic relationship with *F. nucleatum* and *P. gingivalis via* metabolites (Periasamy and Kolenbrander, 2009; Kuboniwa et al., 2009b; Sakanaka et al., 2015). On the other hand, during formation of cariogenic biofilm, cariogenic bacteria represented by the major strain *Streptococcus mutans* become dominant due to multiple factors related to lifestyle and a diet rich in sugars (Selwitz et al., 2007; Gross et al., 2012). In both cases, oral streptococci, which are early colonizers, play a critical role.

Recent studies have shown phenotypic and metabolic heterogeneity in related key species, including *Streptococcus oralis*, a typical oral commensal bacterium, *S. mutans*, a cariogenic bacterium, and *S. gordonii*, which functions as an accessory pathogen in periodontopathic biofilm (Periasamy and Kolenbrander, 2010; Hajishengallis, 2015; Thurnheer and Belibasakis, 2018). Furthermore, other reports have shown competitive relationships between these oral streptococci, as *S. mutans* was found to inhibit the growth of *S. gordonii* by producing the bacteriocin mutacin and *S. gordonii* inhibited mutacin production by producing the bacteriocin challisin (Kreth et al., 2011). Additionally, because of the high acid resistance of *S. mutans*, it has shown better survival in low pH biofilm as compared to acid-sensitive streptococci (Kreth et al., 2011). On the other hand, *S. mutans* is highly sensitive to hydrogen peroxide produced by *S. gordonii* and *S. oralis* (Kreth et al., 2008; Redanz et al., 2018).

Presently, understanding of how to control these species remains limited, though that is considered essential for maintaining oral health. In oral streptococci, glycolysis is a crucial metabolic pathway for energy acquisition and production of organic acids, thus xylitol, a sugar alcohol, is widely used as a sugar substitute to reduce caries risk, because it cannot be metabolized by oral streptococci (L, 1995; Makinen, 2010; Milgrom et al., 2012). However, the preventive effect of xylitol is inhibited by other monosaccharides (Gauthier et al., 1984), while it has also been shown to have side effects in the gastrointestinal tract (Oku and Nakamura, 2007).

D-tagatose is a ketohexose, 4-epimer of fructose that has lower calories and a reduced glycemic effect as compared to sucrose (Donner et al., 1999; Oh, 2007; Guerrero-Wyss et al., 2018; Roy et al., 2018). It has been reported that D-tagatose showed effects on the metabolome of pig intestinal microflora, especially flora in the cecum and colon, and caused increases in butyric acid and valeric acid (Lærke et al., 2000). Other reports have also shown species-specific growth inhibitory effects on *Phytophthora* spp. by D-tagatose (Chahed et al., 2020; Corneo et al., 2021). Based on these findings, this artificial sweetener is expected to be applied in various contexts, such as diabetes management, pesticides, and prebiotics for the intestines. Additionally, D-tagatose has been reported to inhibit the growth of *S. mutans* (Sawada et al., 2015; Hasibul et al., 2018), though there are few studies regarding its effects on other types of oral bacteria. Furthermore, no known reports of comprehensive analyses of tagatose-induced dynamic changes in oral bacteria using metabolomics and transcriptomics have been presented.

In the present study, metabolomic profiling of human saliva samples showed that D-tagatose was associated with good oral hygiene. Furthermore, results of *in vitro* examinations of the effects of D-tagatose on physiology, as well as transcriptomic and metabolomic profiles of the oral streptococci *S. mutans*, *S. gordonii*, and *S. oralis* highlight its potential use as an oral prebiotic for altering the metabolism of those oral streptococci.

## Materials and Methods

### Saliva Metabolome

Data for the present study were obtained by reanalysis of raw data from our previous study (Kuboniwa et al., 2016) using MS-DIAL version 3.90 (Tsugawa et al., 2015). Briefly, that previous study was conducted from 2013 to 2014 with approval from the Osaka University Research Ethics Committee, and in accordance with the principles of the Helsinki Declaration and STROBE guidelines for human observational studies. Nineteen participants provided written informed consent prior to enrolment. Each subject was asked to refrain from eating, drinking, or toothbrushing for at least one hour prior to undergoing the procedures. Five calibrated and licensed dentists evaluated plaque accumulation at four sites per tooth for all present, and the average per tooth was used for the total score, which was defined as modified plaque index (mPlI) (Sakanaka et al., 2017). mPlI was not obtained in that manner for one of the subjects, and all participants except for this subject were asked to expectorate unstimulated whole saliva over a 10-minute period into a 50-mL tube (Corning, Corning, NY, USA) that was kept on ice. Subsequently, the 18 samples were frozen with liquid nitrogen and stored at −80°C until analysis. Metabolomics raw data were obtained using gas chromatography coupled with mass spectrometry (GCMS-QP2010 Ultra, Shimazu).

### Bacterial Strains and Growth Conditions

The strains used in this study are listed in **Table 1**. All were grown statically at 37°C under aerobic conditions in brain-heart infusion broth (BHI; Becton, Dickinson and Company, Franklin Lakes, NJ, USA) or, when necessary, in chemically defined medium (CDM), developed according to a previously reported method (Loo et al., 2000). Briefly, CDM consisting of 58 mM K_2_HPO_4_, 15 mM KH_2_PO_4_, 10 mM (NH_4_)_2_SO_4_, 35 mM NaCl, 0.2% casamino acids, and 100 μM MnCl_2_ 4H_2_O (pH 7.4) was supplemented with filter-sterilized vitamins (0.04 mM nicotinic acid, 0.1 mM pyridoxine HCl, 0.01 mM pantothenic acid, 1 μM riboflavin, 0.3 μM thiamin HCl, 0.05 μM D-biotin), amino acids (4 mM L-glutamic acid, 1 mM L-arginine HCl, 1.3 mM L-cysteine HCl, 0.1 mM L-tryptophan), and MgSO_4_ 7H_2_O (2 mM).

**Table 1:** Bacterial strains used in this study.

Strains	Description	Source or Ref.
*Streptococcus gordonii* DL1 Challis	Wild type	Pakura and Walczak
*Streptococcus mutans* OMZ175	Wild type	B. Guggenheim
*Streptococcus mutans* UA159	Wild type	University of Alabama
*Streptococcus oralis* ATCC9811	Wild type	American Type Culture Collection

### Saliva Preparation

Saliva was collected in a sterile centrifuge tube on ice from six healthy donors and pooled, as described previously (Kuboniwa et al., 2009a). Dithiothreitol (Sigma-Aldrich, St. Louis, MO) was added to a 2.5 mM final concentration, then the saliva was gently stirred on ice for 10 minutes and centrifuged at 3,000 × g for 20 minutes at 4°C. The clarified saliva supernatant was decanted, 3 volumes of distilled water was added, and the 25% saliva was filtered through a 0.20 μm pore size filter and frozen in 40 ml aliquots. Immediately prior to use, the sterile saliva was thawed at 37°C; the slight precipitate was pelleted at 1,430 × g for 5 min, and the clear 25% saliva supernatant was used in experiments.

### Biofilm Assay

*S. oralis* ATCC9811, *S. mutans* OMZ175, UA159, and *S. gordonii* Challis DL1 cells that reached the late logarithmic growth phase were collected, then subjected to centrifugation (7,670 × g, 4°C, 7 minutes). Cells (10^8^ CFU) were then stained with hexidium iodide (HI; Molecular Probes, Carlsbad, CA, USA) and cultured in CDM containing 0.8% D-glucose, sucrose, D-tagatose, or no sugar at 37°C under aerobic conditions for 24 hours using the eight-well LAB-TEK Chamber Slide System (Nalge Nunc International, Naperville, IL, USA) after coating with 25% sterile human saliva. Biofilm formed on the bottom surface of each chamber was observed using a confocal laser scanning microscope [(CLSM), TCS SP8; Leica Microsystems GmbH, Wetzlar, Germany]. Images of 10 random fields of view in each chamber were obtained, then analyzed using the Imaris 7.0.1 software package (BitplaneAG, Zurich, Switzerland).

### Growth Study

*S. oralis* ATCC9811, *S. mutans* OMZ175, UA159, and *S. gordonii* Challis DL1 cells (10^8^ CFU) were cultured under aerobic conditions at 37°C in BHI without or with D-tagatose at a concentration of 0.1%, 0.5%, 1%, 5%, or 10%. Similarly, the bacteria were cultured in CDM containing 0.2% D-glucose without or with D-tagatose at a concentration of 1% or 5%. Optical density at 600 nm (OD_600_) was determined using an ultraviolet-visible spectrophotometer (UV-1600; Shimadzu Corporation, Kyoto, Japan). To investigate whether D-tagatose is utilized by *S. oralis*, *S. mutans*, and/or *S. gordonii*, aerobic cultures were performed at 37°C in CDM without or with 0.8% D-tagatose or D-glucose.

### Viability Assay

Bacterial cells were cultured at 37°C for 48 hours under aerobic conditions in CDM containing 0.2% D-glucose without or with D-tagatose at a concentration of 1% or 5%, then stained using a LIVE/DEAD^®^ BacLight™ Bacterial Viability Kit (Molecular Probes, Eugene, OR), according to the manufacturer’s instructions. Stained cells were observed using a multi-label counter (Wallac 1420 ARVO MX; Perkin Elmer, Waltham, Massachusetts) (excitation wavelength 485 nm, emission wavelength 535 nm). Viable cell counts were calculated based on a calibration curve.

### Extracellular Polysaccharide (EPS) Production Assay

*S. mutans* OMZ175 was cultured under aerobic conditions at 37°C for 48 hours in CDM containing 0.2% D-glucose without or with 1% or 5% D-tagatose. The cells were then collected by centrifugation (7,670 × g, 4°C, 7 minutes) and washed with 10 mM phosphate-buffered saline (PBS, pH 7.4). EPS was stained with concanavalin A (Con A) and wheat germ agglutinin (WGA), and bacterial cells with HI, which were then washed and suspended in PBS. Bacterial suspensions (OD_600_ = 4.0) in 10 random fields were observed with CLSM. Obtained images were analyzed using the Imaris 7.0.1 software package.

### Intracellular Metabolite Measurement

*S. oralis* ATCC9811, *S. mutans* UA159, and *S. gordonii* Challis DL1 were initially adjusted to an OD_600_ of 0.8, then grown aerobically at 37°C in CDM containing 0.2% D-glucose without or with 1% or 5% D-tagatose. After three hours of incubation, bacterial cells were harvested by centrifugation (7,670 × g, 4°C, 7 minutes), washed with 1 ml of ultrapure water, and immediately frozen by liquid nitrogen. Ribitol was added as an internal standard for normalization, then 1.3 ml of acetonitrile was added to remove protein. After thoroughly mixing the samples, centrifugation was performed at 20,630 × g for 3 minutes at 4°C. The upper 1.6 ml was evaporated in a centrifugal concentrator for 120 minutes at room temperature and freeze-dried overnight. One hundred μl of 20 mg/mL methoxyamine hydrochloride in pyridine and 50 μl N-methyl-N-trimethylsilyltrifluoroacetamide (MSTFA, Sigma-Aldrich) were added to the samples as derivatizing agents, then they were thoroughly mixed and centrifuged at 15,490 × g for 3 minutes at 25°C, after which the upper 50 μl of each sample was transferred to a vial. Measurement of extracted metabolites was performed using a GC-MS/MS-TQ8040 device (Shimadzu). Obtained GC/MS data were analyzed with an ABF converter (https://www.reifycs.com/AbfConverter/index.html) and the MS-DIAL software package, ver. 4.60 (http://prime.psc.riken.jp/Metabolomics_Software/MS-DIAL/index.html).

### RNA Extraction and Sequencing

Triplicate samples used for extracting total RNA were cultured for 30 minutes under the same conditions as for those subjected to metabolomics analysis. Bacteria harvested by centrifugation were immediately frozen and homogenized with zirconia beads at 1,500 rpm for 5 minutes at 4°C with use of a ShakeMaster NEO (BioMedical Science, Tokyo, Japan). Total RNA was isolated using TRIzol reagent (Life Technologies, Inc.) and an RNeasy kit (Qiagen). RNA quality was evaluated based on test concentration (Nanodrop 1000), agarose gel electrophoresis (AGE), and BioAnalizer results. Quality-controlled RNA samples were reversed into cDNA and then used to construct a sequencing library, sequenced using an Illumina NovaSeq 6000 by Rhelixa Co., Ltd. (Tokyo, Japan).

### Statistical Analysis

All pairwise comparisons of groups were performed using a two-tailed t-test. Comparisons of multiple groups, except for growth curve analyses, were performed using one-way analysis of variance with *post hoc* paired comparisons conducted with Dunnett’s test. All statistical analyses were done with SPSS version 26 (IBM Japan, Tokyo, Japan), with a *p* value of 0.05 considered to be statistically significant. Metabolite set enrichment analysis (MSEA) was performed as previously described (Sakanaka et al., 2017). Differentially expressed genes (DEGs) were identified using the Bioconductor package edgeR. Gene Ontology (GO) enrichment analysis of DEGs was performed using the Database for Annotation, Visualization and Integrated Discovery (DAVID) of the NIH.

## Results

### Saliva Metabolome

**Figure 1:**
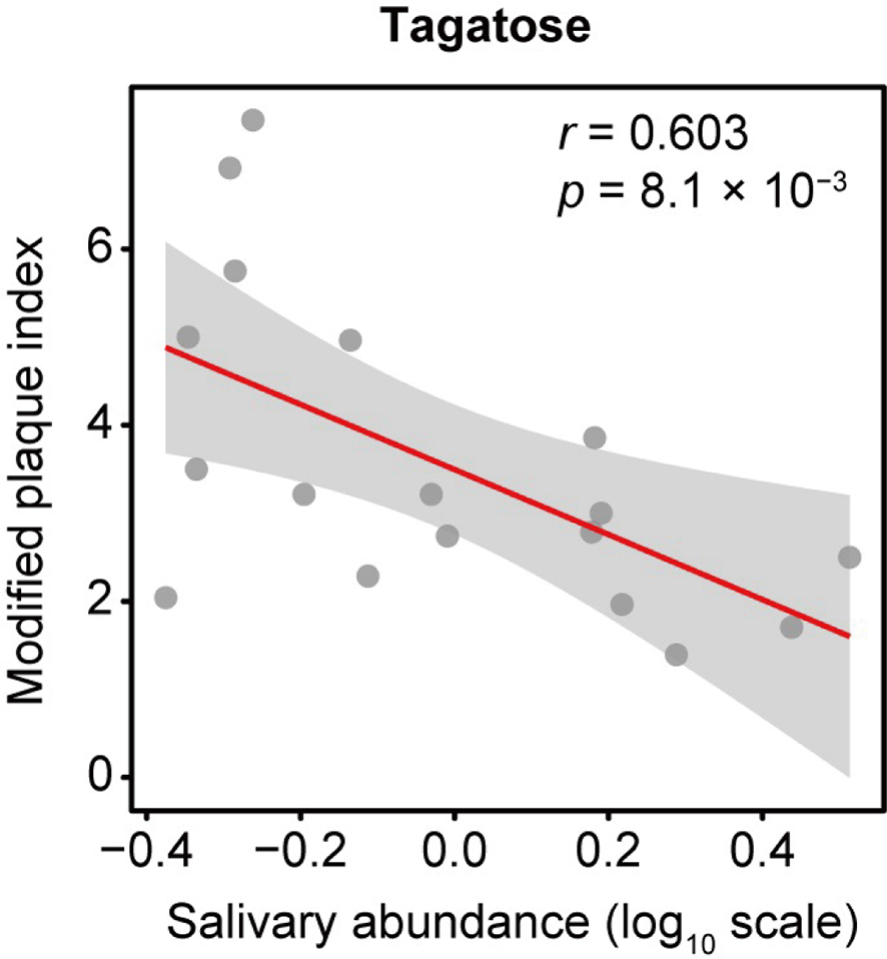
Association of salivary tagatose with modified plaque index (mPlI). The correlation between D-tagatose and mPlI, considered to be an indicator of oral hygiene status, was evaluated in 18 subjects for whom mPlI data were available. D-tagatose was found to be significantly negatively correlated with mPlI (Spearman’s correlation coefficient; r = -0.603, p = 0.008; ). It is suggested that tagatose may play a role in maintaining a low level of plaque and thus good hygiene in the oral cavity.

Data analyzed from our previous study (Kuboniwa et al., 2016) using the new software package MS-DIAL showed 114 annotated metabolites, with D-tagatose among them. The correlation between D-tagatose and mPlI, considered to be an indicator of oral hygiene status, was evaluated in 18 subjects for whom mPlI results were available, which showed that D-tagatose was significantly negatively correlated with mPlI (Spearman’s correlation coefficient; *r* = -0.603, *p* = 0.008; **Figure 1**). This suggested that tagatose may play a role in maintaining a low level of plaque and thus promotes good hygiene in the oral cavity.

### Effects of D-Tagatose on Planktonic Growth

**Figure 2:**
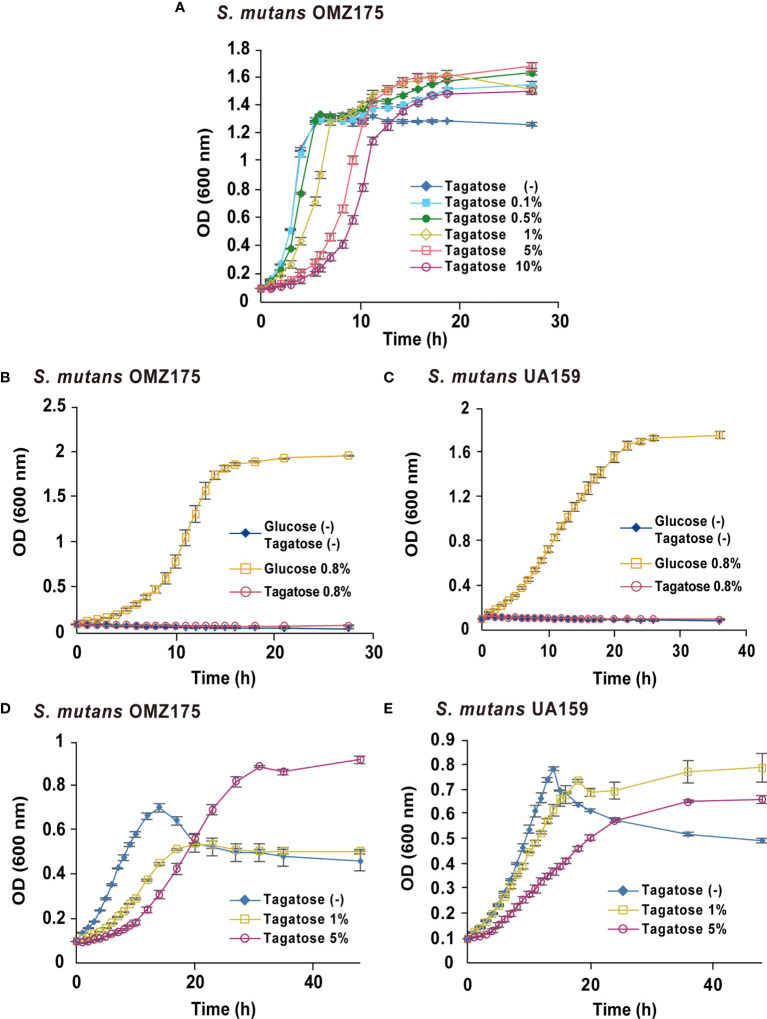
Bacterial growth of *S. mutans*. All data are shown as the mean ± S.D. of triplicate experiments. **(A)** Growth of *S. mutans* OMZ175 in BHI containing various concentrations of D-tagatose. **(B, D)** Growth of *S. mutans* OMZ175 and UA159 in CDM containing no sugar, 0.8% D-glucose, or 0.8% D-Tagatose. **(C, E)** Growth of *S. mutans* OMZ175 and UA159 in CDM containing 0.2% D-glucose with 0%, 1%, or 5% D-tagatose.

**Figure 3:**
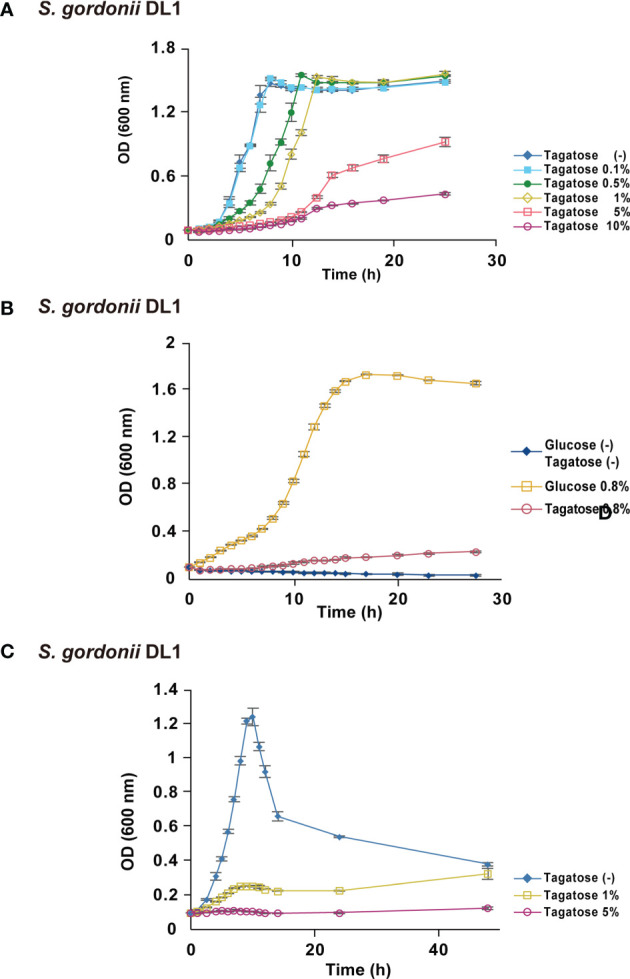
Bacterial growth of *S. gordonii*. All data are shown as the mean ± S.D. of triplicate experiments. **(A)** Growth of *S. gordonii* in BHI containing various concentrations of D-tagatose. **(B)** Growth of *S. gordonii* in CDM containing no sugar, 0.8% D-glucose, or 0.8% D-Tagatose. **(C)** Growth of *S. gordonii* in CDM containing 0.2% D-glucose with 0%, 1%, or 5% D-tagatose.

**Figure 4:**
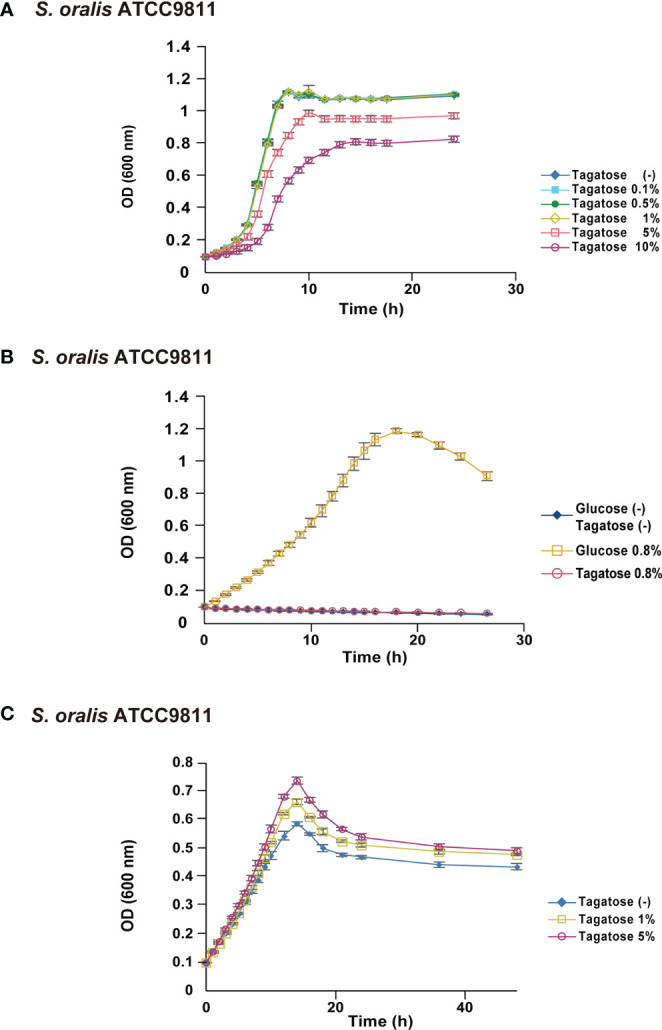
Bacterial growth of *S. oralis*. All data are shown as the mean ± S.D. of triplicate experiments. **(A)** Growth of *S. oralis* in BHI containing various concentrations of D-tagatose. **(B)** Growth of *S. oralis* in CDM containing no sugar, 0.8% D-glucose, or 0.8% D-Tagatose. **(C)** Growth of *S. oralis* in CDM containing 0.2% D-glucose with 0%, 1%, or 5% D-tagatose.

To focus on the potential of D-tagatose as a prebiotic, its effects on the physiology of multiple oral streptococci were investigated. First, growth of *S. gordonii*, *S. mutans*, and *S. oralis* in BHI containing D-tagatose was examined, which showed that the lag phase of growth of *S. gordonii* and *S. mutans* was prolonged in the presence of D-tagatose at a concentration of 0.5% or higher (**Figures 2A**, **3A**). On the other hand, *S. oralis* growth was unaffected by D-tagatose at a concentration of 1% or lower. However, growth was inhibited 5% and 10% D-tagatose, though inhibition of *S. oralis* growth was limited as compared to that seen with *S. gordonii* and *S. mutans* (**Figure 4A**).

Growth of these species in the presence of D-tagatose as the sole energy source was examined. *S. gordonii*, *S. mutans*, and *S. oralis* growth was not supported in CDM containing 0.8% D-tagatose instead of 0.8% D-glucose (**Figures 2B, D**, **3B**, **4B**), same as in CDM with no energy source, indicating that D-tagatose is not used by these species as an energy source. When cultured in CDM containing D-glucose, D-tagatose inhibited the growth of *S. gordonii* and *S. mutans* cells in the exponential phase, though the final OD_600_ of *S. mutans* was elevated (**Figures 2C, E**, **3C**), while no such inhibitory effect on growth of *S. oralis* was observed (**Figure 4C**). These results suggested that D-tagatose selectively inhibits planktonic growth of *S. gordonii* and *S. mutans*.

### Effects of D-Tagatose on Bacterial Viability

**Figure 5:**
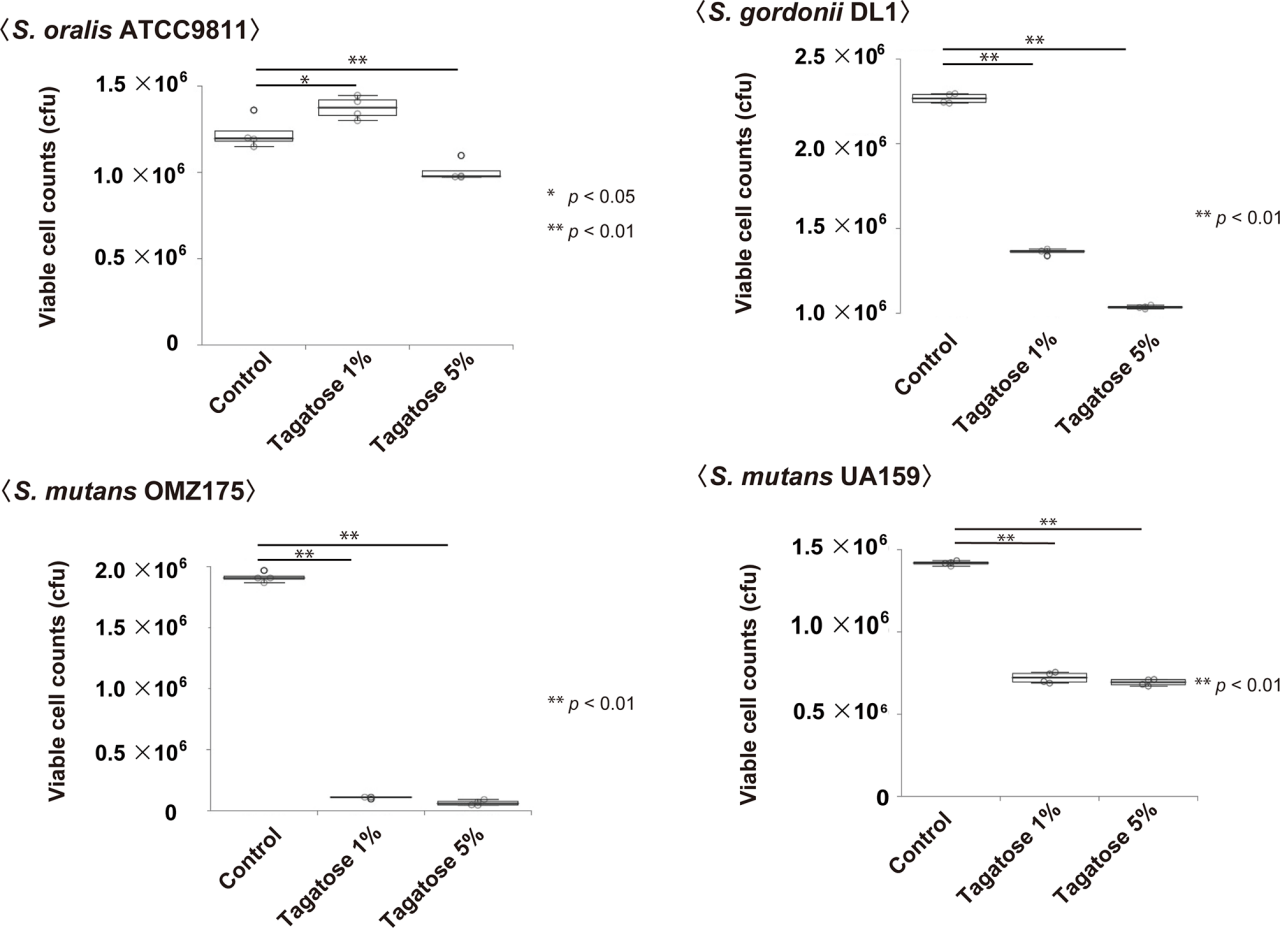
Effects on viable cells of *S. mutans*, *S. gordonii*, and *S. oralis*. Cells were incubated in CDM containing 0.2% glucose with 0%, 1%, or 5% tagatose for 48 hours. Statistical differences were calculated using Dunnett’s test. *p < 0.05, **p < 0.01.

Assays of bacterial viability in the presence of D-tagatose showed significantly decreased numbers of viable *S. gordonii* and *S. mutans* cells, which occurred in a dose-dependent manner (**Figure 5**). In contrast, the number of viable cells of *S. oralis* was increased when incubated with 1% D-tagatose in CDM, while there was only a negligible decrease in the presence of 5% D-tagatose (**Figure 5**). Accordingly, D-tagatose was shown to selectively suppress the viability of *S. gordonii* and *S. mutans*, and had very limited effects on *S. oralis*.

### Effects of D-Tagatose on Production of EPS in *S. mutans*

**Figure 6:**
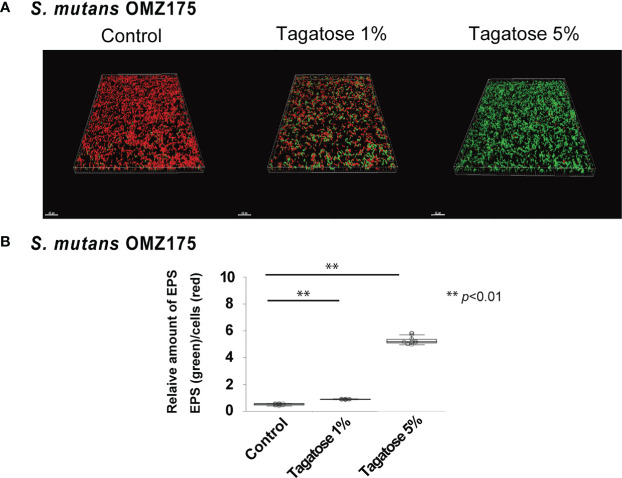
Effects of D-tagatose on *S. mutans* EPS production. **(A)** Representative CLSM images showing typical architecture of planktonic cells (red) and EPS (green) following reconstruction with Imaris software. **(B)** Relative amounts of EPS to cells. Ten fields per sample were randomly recorded with CLSM and the relative amount of EPS was calculated using IMARIS software. Control, without tagatose. **P < 0.01.

To examine the increase in final OD_600_ of *S. mutans* in the presence of both glucose and tagatose, EPS production assays were performed. Since not only viable cell count but also extracellular products could have effects on OD_600_ in UV-visible spectrophotometer readings (Takahashi et al., 2004), EPS production by *S. mutans* under a D-tagatose stress condition was confirmed. Confocal imaging results showed that the ratio of EPS/cell was altered by treatment with D-tagatose (**Figure 6A**), as the treated groups exhibited a significant increase in relative quantity of EPS as compared with the control, in a dose-dependent manner (**Figure 6B**). These findings indicated that tagatose impairs the viability of *S. mutans*, though accelerates its EPS production in the presence of glucose, corresponding to the change in final OD_600_ seen with D-tagatose addition.

### Effects of D-Tagatose on Biofilm Formation

**Figure 7:**
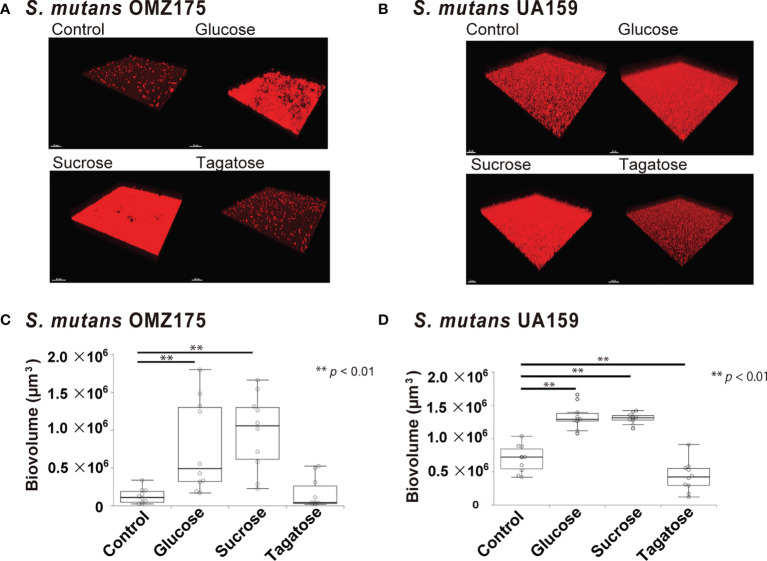
Effects of D-Tagatose on *S. mutans* biofilm formation. **(A, C)** Representative CLSM images showing typical architecture of biofilms following reconstruction with Imaris software. *S. mutans* cells were stained with hexidium iodide (red), and incubated with saccharides or nothing was added. **(B, D)** Biovolume analysis of *S. mutans*. Ten fields per sample were randomly recorded with CLSM and *S. mutans* biovolume was quantified using IMARIS software. Control, containing no sugar. **P < 0.01.

**Figure 8:**
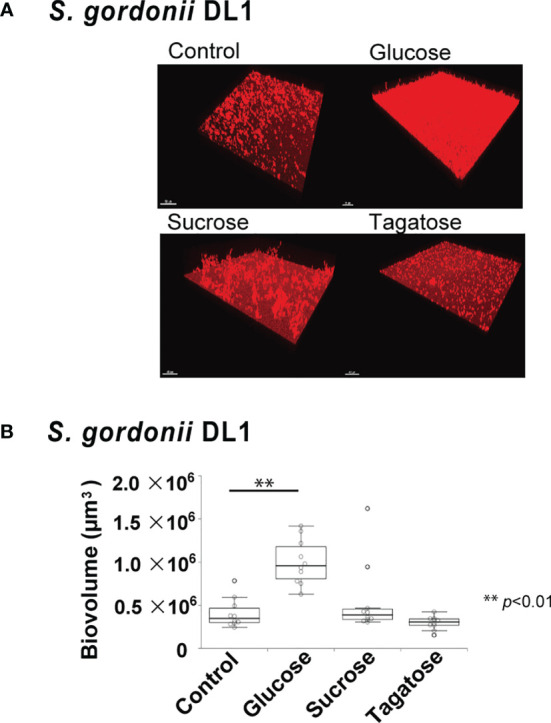
Effects of D-Tagatose on *S. gordonii* biofilm formation. **(A)** Representative CLSM images showing typical architecture of biofilms following reconstruction with Imaris software. *S. gordonii* cells were stained with hexidium iodide (red), and incubated with saccharides or nothing was added. **(B)** Biovolume analysis of *S. gordonii*. Ten fields per sample were randomly recorded with CLSM and *S. gordonii* biovolume was quantified using IMARIS software. Control, containing no sugar. **P < 0.01.

**Figure 9:**
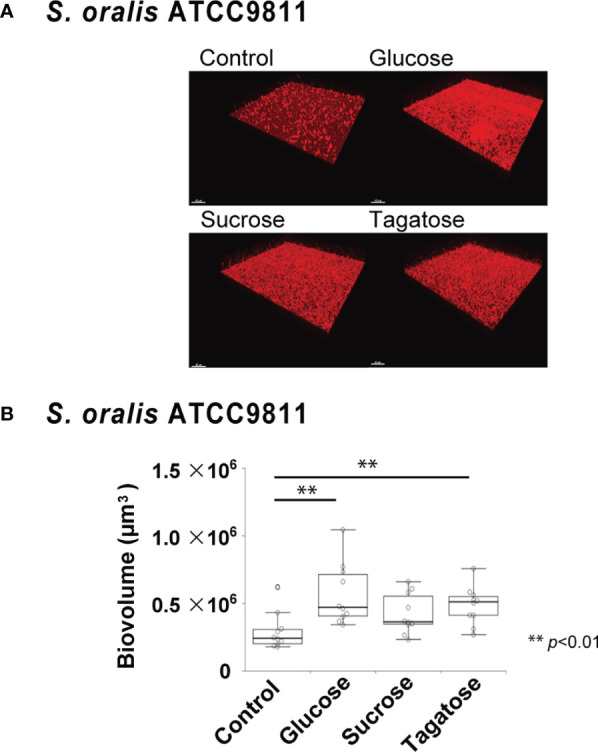
Effects of D-Tagatose on *S. oralis* biofilm formation. **(A)** Representative CLSM images showing typical architecture of biofilms following reconstruction with Imaris software. *S. oralis* cells were stained with hexidium iodide (red), and incubated with saccharides or nothing was added. **(B)** Biovolume analysis of *S. oralis*. Ten fields per sample were randomly recorded with CLSM and *S. oralis* biovolume was quantified using IMARIS software. Control, containing no sugar. **P < 0.01.

The effects of D-tagatose on biofilm formation by the tested strains were examined using biofilm assay results as well as examinations of confocal imaging data of biofilms cultured in CDM containing glucose, sucrose, or tagatose as the sole carbon source. When cultured in CDM containing D-tagatose, *S. gordonii* and *S. mutans* each showed negligible biofilm formation, whereas significantly increased biofilm formation was noted when grown in CDM containing D-glucose (**Figures 7**, **8**). Treatment with sucrose resulted in robust biofilm formation by *S. mutans*, whereas biofilms formed by *S. gordonii* were relatively fragile with some detached cells observed. Interestingly, *S. oralis* formed significantly increased biofilm not only when the cells were treated with D-glucose but also when exposed to D-tagatose (**Figure 9**), suggesting that this strain may have a capacity for utilization of D-tagatose, in contrast to *S. gordonii* and *S. mutans*.

### RNA-Seq Analysis

To gain insight into the species-specific effects of D-tagatose, RNA-seq analysis of the examined species in the presence of tagatose was performed. Bacterial cells were incubated in CDM containing D-tagatose for 30 minutes, then total RNA was extracted from each sample. The results showed detection of 199 *S. mutans* genes (116 up-regulated, 83 down-regulated), 38 *S. gordonii* genes (35 up-regulated, three down-regulated), and 12 *S. oralis* genes (four up-regulated, eight down-regulated) as DEGs (FDR <0.05, |log_2_(fold change)| >1) when the cells were incubated with 5% D-tagatose. Volcano plots of DEGs also showed that D-tagatose altered the transcriptome profiles of each of these three streptococci (**Figure S1**).

**Figure 10:**
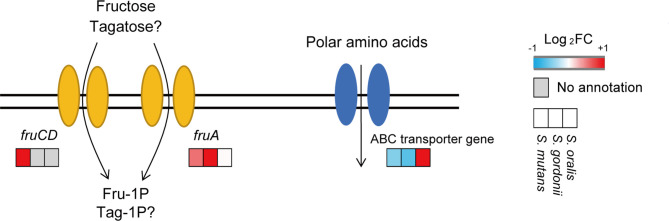
Expressions of transporter genes. Gene expression levels of fructose-specific PTS and amino acid ABC transporters are shown. The three squares show Log_2_-transformed fold change values for tagatose-incubated cells calculated as compared to the control *S. mutans* (left square), *S. gordonii* (center square), and *S. oralis* (right square), according to a reported color scale.

DEGs of *S. mutans* and *S. gordonii* when incubated with 5% D-tagatose are listed in **Table S1**. GO analysis of *S. mutans* showed that genes related to the histidine biosynthetic and lactose catabolic processes *via* D-tagatose-6-phosphate were up-regulated, whereas those related to the phosphoenolpyruvate-dependent sugar phosphotransferase system (PTS) were down-regulated (*p <*0.05) (**Table S2**). Also, GO analysis of *S. gordonii* showed that genes related to lactose metabolism, galactose catabolism, and PTS were up-regulated (*p <*0.05) (**Table S2** and **Figure S1**), while KEGG pathway analysis revealed that fructose-PTS genes (SMU_114,115, SGO_1111-1113) were up-regulated in both *S. mutans* and *S. gordonii* (**Figure S1**). These changes in gene expression indicate that *S. mutans* and *S. gordonii* take up D-tagatose *via* fructose-PTS (**Figure 10**), and showed that variations in gene expression for these two species are similar.

*S. oralis* DEGs are also presented in **Table S1**. There was no information in DAVID regarding *S. oralis* ATCC9811, thus GO analysis was not performed. The expression of fructose-specific PTS genes in *S. oralis* was not altered by 5% D-tagatose (**Figure S1**). On the other hand, that changed gene expressions related to the ATP binding cassette (ABC) transporter. Additionally, a polar amino acid ABC transporter permease gene (SOR_1115) was up-regulated (**Figure 10**), while sugar ABC transporter genes (SOR_0121, 1899) were down-regulated.

### Alteration of Metabolomic Profile

**Figure 11:**
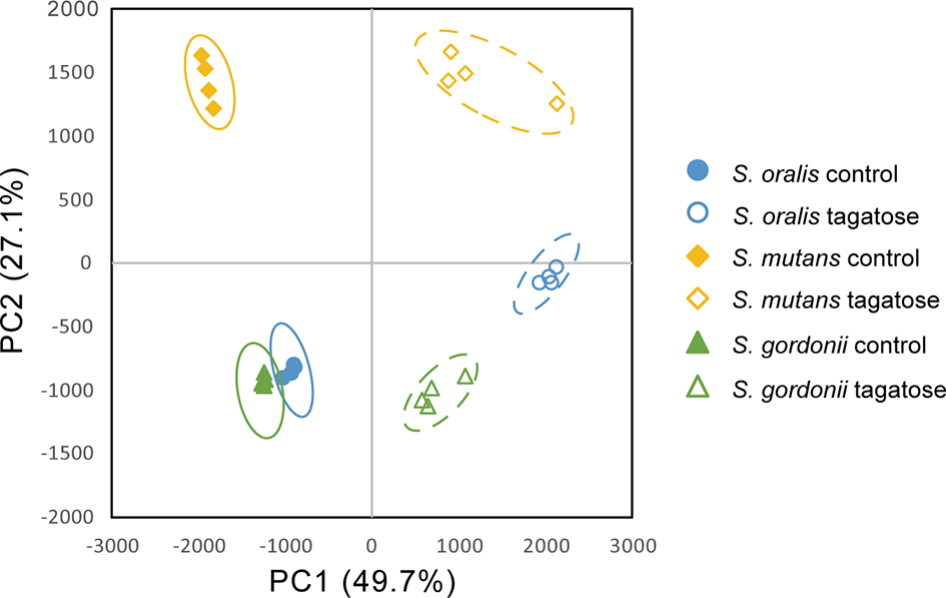
Principal component analysis of intracellular metabolome of *S. gordonii*, *S. mutans*, and *S. oralis*. Score plots for PC1 and PC2 are shown.

**Figure 12:**
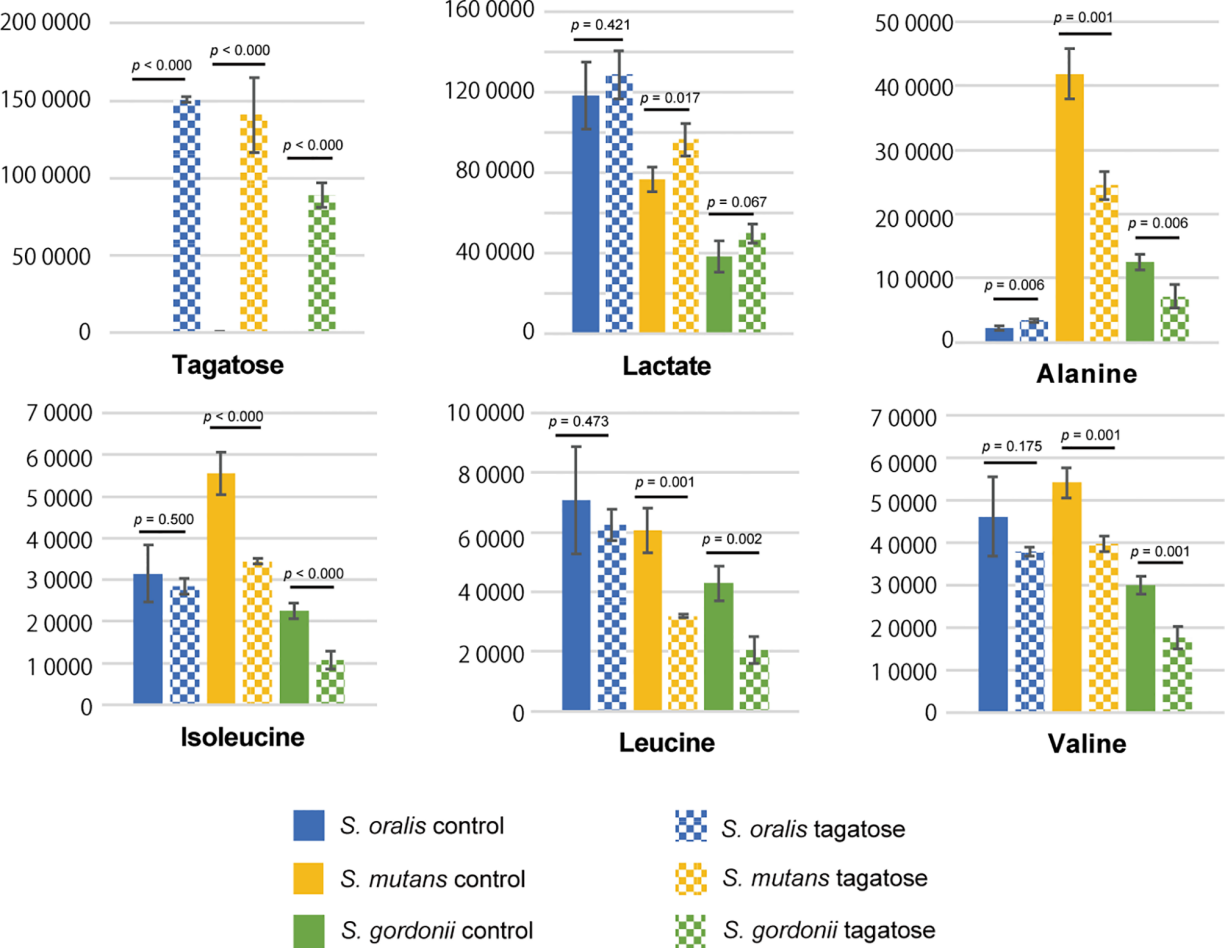
Contents of tagatose and downstream metabolites from pyruvate. Metabolites were extracted from *S. mutans*, *S. gordonii*, and *S. oralis* cells after three hours of incubation in CDM with or without tagatose, followed by determination of those levels using GC-MS. To minimize the effects of different growth stages, the OD_600_ value was set to 0.8 before starting the culture. Bars represent the mean ± S.D. (n = 4). P-values were determined with Student’s *t*-test.

Metabolomic analysis was also performed to observe the effects of changes in gene expression profiles on the tested strains. Principal component analysis illustrated differences in intracellular metabolite profiles between the strains with or without D-tagatose (**Figure 11**). MSEA was done to explore metabolic pathways altered by D-tagatose, and a comparison of those results between the control and D-tagatose groups showed alterations in glycolysis and alanine metabolism (**Table S3**). Glycolysis is a series of metabolism reactions in which glucose is oxidized to pyruvate, then converted to organic acids, alanine, branched-chain amino acids (BCAAs), and others. A variety of factors are involved in reprogramming the glycolysis pathway, for example, shift of lactate fermentation to BCAA production for pH homeostasis (Matsui and Cvitkovitch, 2010). Five metabolites were detected as fermentation products in *S. oralis*, *S. mutans*, and *S. gordonii* (lactate, alanine, leucine, isoleucine, valine) as the result of glycolysis (**Figure 12**). D-tagatose significantly increased lactate in *S. mutans*, while that showed an increasing tendency in *S. gordonii* (*p* = 0.067). In contrast, D-tagatose did not increase lactate in *S. oralis* (*p* = 0.421). Alanine was significantly decreased in *S. mutans* and *S. gordonii*, while it was increased in *S. oralis*. Furthermore, D-tagatose significantly decreased leucine, isoleucine, and valine in *S. mutans* and *S. gordonii*, but not in *S. oralis*. Together, these results suggested that D-tagatose has effects on energy production *via* the PTS system, as well as BCAAs and alanine production *via* the pyruvate oxidation pathway, leading to disruption of metabolic homeostasis.

## Discussion

Results of this study indicate that D-tagatose causes species-specific transcriptomic and metabolomic changes in *S. mutans*, *S. gordonii*, and *S. oralis*, with different inhibitory properties demonstrated with each strain. D-tagatose is a non-cariogenic sugar known to suppress growth of *S. mutans*. The present findings provide further evidence in that regard, as transcriptome and metabolome changes in oral streptococci including *S. mutans* caused by D-tagatose have not been previously reported. The tested strains did not grow in the presence of D-tagatose, though its uptake by each strain was demonstrated. The diverse transcriptome variations due to D-tagatose noted among the test strains suggests that the mechanisms of its uptake are different.

Fructose-specific PTS, whose genes are up-regulated by D-tagatose in *S. mutans* and *S. gordonii*, consumes ATP and converts fructose to fructose-1-phosphate, and *S. mutans* has two operons that encode components of fructose-specific PTS (FruI, FruCD) (Wen et al., 2001). D-tagatose was found to increase the expression levels of *fluI* and *fruCD* in *S. mutans* by about 1.45 and 33-34 fold, respectively, while *fruA*, a fructose-specific PTS gene encoded by *S. gordonii* and *S. oralis*, was up-regulated by approximately 11-fold in *S. gordonii*, but negligibly in *S. oralis*. Transcription of *fruI* and *fruA* is allosterically controlled by FruR, a DeoR family transcriptional regulator, and that transcription is repressed in the absence of the inducer fructose-1-phosphate (Yoon et al., 2021). No direct uptake activity of D-tagatose *via* PTS was detected in the present study, though our other results suggested that D-tagatose phosphorylated by FruI, FruA, or FruCD may accumulate in *S. mutans* and *S. gordonii*, and then function as an inducer of fructose-specific PTS gene expression. Accumulation of phosphorylated saccharides is known to cause sugar-phosphate stress, which results in harmful effects towards gram-negative organisms (Vanderpool and Gottesman, 2004; Maleki et al., 2017).

Accumulation of fructose-1-phosphate in *S. mutans* increases cell lysis and envelope DNA release (Zeng and Burne, 2019). Fructose-specific PTS is also involved in xylitol uptake (Wen et al., 2001). Xylitol is non-acidogenic sugar alcohol known for its inhibitory effect on *S. mutans*, which takes it up *via* fructose-specific PTS, causing accumulation of xylitol-5-phosphate that cannot be metabolized, resulting in triggering of sugar-phosphate stress (L, 1995; Kakuta et al., 2003). These facts support the speculation that *S. mutans* and *S. gordonii* uptake D-tagatose *via* fructose-specific PTS, and accumulate phosphorylate D-tagatose.

The present GO analysis results showed that the D-tagatose-6-phosphate pathway, a branch of galactose metabolism, was activated in *S. mutans* and *S. gordonii*. Transcription of the lactose operon (*lacABCD*), a D-tagatose-6-phosphate pathway gene cluster, is allosterically regulated by LacR, a DeoR family transcriptional regulator, which becomes inactivated by the presence of D-tagatose-6-phosphate (Rooijen et al., 1993). Thus, these results suggest that *S. mutans* and *S. gordonii* convert D-tagatose to D-tagatose-6-phosphate. However, no growth of *S. mutans* or *S. gordonii* was seen in the present of D-tagatose, even though both showed a metabolic pathway to obtain energy from D-tagatose-6-phosphate. Additional research is needed to assess whether *S. mutans* and *S. gordonii* acquire ATP from D-tagatose.

Analyses of metabolomics data indicate that changes in the metabolomic profile of glycolytic end-metabolites due to D-tagatose treatment varied among the examined bacterial species. Amino acid pools such as alanine and BCAAs were decreased in *S. mutans* and *S. gordonii*, but did not in *S. oralis*. In support of those findings, previous reports have noted that D-tagatose caused species-specific growth inhibition of *Phytophthora* spp. and decreased amino acid content in *Phytophthora infestans* (Chahed et al., 2020; Chahed et al., 2021). In *S. oralis*, the polar amino acid transporter gene (SOR_1115) was up-regulated, whereas SMU_1179c and SGO_1037, orthologous genes of SOR_1115, were down-regulated. These gene expression changes may trigger changes in BCAAs and alanine pools, since glutamate and its substrate glutamine are polar amino acids, and streptococci utilize glutamate for synthesis of alanine and BCAAs. Alanine and glutamate are utilized for synthesis of peptidoglycan constituting cell walls (Chapot-Chartier and Kulakauskas, 2014; Huang et al., 2020), while BCAAs are used for synthesis of membrane branched-chain fatty acids (Kaneda, 1963; Kaneda, 1977; Santiago et al., 2012). In addition, BCAAs have been shown to activate the global regulator CodY for transcriptional reprogramming (Sonenshein, 2007; Santiago et al., 2012). Therefore, BCAAs and alanine are essential for cell replication and adaptation to environmental changes. On the other hand, a previous report indicated that accumulation of organic acids lowers intracellular pH and suppresses glycolysis (Dashper and Reynolds, 2000). Among the present test strains, only *S. oralis* has the L-lactate oxidase gene (*lctO*) and can oxidize lactate to pyruvate under aerobic conditions. As for *Streptococcus pneumoniae*, it has been reported that LctO and pyruvate oxidase (SpxB) may work collaboratively to efficiently obtain ATP (Taniai et al., 2008). Thus, it is possible that such an orchestrated action also contributes to avoiding accumulation of lactate in *S. oralis*. In addition, in consideration of the findings showing alanine increase by D-tagatose in *S. oralis*, it is possible that lactate is reused for alanine synthesis *via* LctO. Therefore, results indicating different metabolome profiles among the tested bacterial species reveal a link to the species-specific inhibitory effect of D-tagatose noted in this study.

Our analyses of clinical saliva samples showed D-tagatose as a substance that characterizes individuals with good oral hygiene. This result appears to be well supported by previous findings indicating that chewing gum containing D-tagatose reduced the number of aerobic and anaerobic bacteria in saliva (Nagamine et al., 2020). D-tagatose is an extremely rare monosaccharide in nature, though can be produced not only chemically, but also biologically using enzymes such as L-arabinose isomerase and D-tagatose 4-epimerase (Kim, 2004; Oh, 2007; Shin et al., 2020). Therefore, the origin of D-tagatose detected in saliva is unknown, though it cannot be denied that it may be derived from a metabolite of oral bacteria. In other words, it is likely that the diversity of the oral microbiome is involved in the presence of D-tagatose in saliva.

In summary, the present study provides evidence that D-tagatose is abundant in the saliva of individuals with good oral health, and the experimental results also demonstrated selectively inhibited growth of the oral pathogens *S. mutans* and *S. gordonii* by D-tagatose. In contrast, the oral commensal *S. oralis* seemed to be negligibly affected, highlighting the potential of use of D-tagatose as an oral prebiotic for manipulating the metabolism of these oral streptococci.

## Data Availability Statement

The data presented in the study are deposited in the DNA Data Bank of Japan Sequence Read Archive repository, accession number DRA012703, https://ddbj.nig.ac.jp/public/ddbj_database/dra/fastq/DRA012/DRA012703/.

## Ethics Statement

The data presented in the study are deposited in the DNA Data Bank of Japan Sequence Read Archive repository, accession number DRA012703.

## Author Contributions

SM contributed to design, data acquisition, analysis, and interpretation, drafted and critically revised the manuscript. MK contributed to conceptions, design, data acquisition, analysis, and interpretation, drafted and critically revised the manuscript. AS contributed to design, data acquisition, analysis, and interpretation, drafted and critically revised the manuscript. EH contributed to design, data acquisition, analysis, and interpretation, critically revised the manuscript. AI and YI contributed to data acquisition, critically revised the manuscript. AA contributed to design, data analysis and interpretation, critically revised the manuscript. All authors gave final approval and agree to be accountable for all aspects of the work.

## Funding

This research is funded by a Japan Society for the Promotion of Science (JSPS) Grant-in-Aid for Challenging and Pioneering Research (grant 20K20393) and JSPS KAKENHI grants (grants 18H04068, 19H03862, 18K17281, 20K18827).

## Conflict of Interest

The authors declare that the research was conducted in the absence of any commercial or financial relationships that could be construed as a potential conflict of interest.

## Publisher’s Note

All claims expressed in this article are solely those of the authors and do not necessarily represent those of their affiliated organizations, or those of the publisher, the editors and the reviewers. Any product that may be evaluated in this article, or claim that may be made by its manufacturer, is not guaranteed or endorsed by the publisher.
