# Supplementary material for: Potential of Prebiotic D-Tagatose for Prevention of Oral Disease
Source: Front Cell Infect Microbiol. 2021 Nov 5;11:767944. doi: 10.3389/fcimb.2021.767944 (PMC8604381; doi:10.3389/fcimb.2021.767944)
Supplement: Supplementary file 1 [file DataSheet_1.pdf]

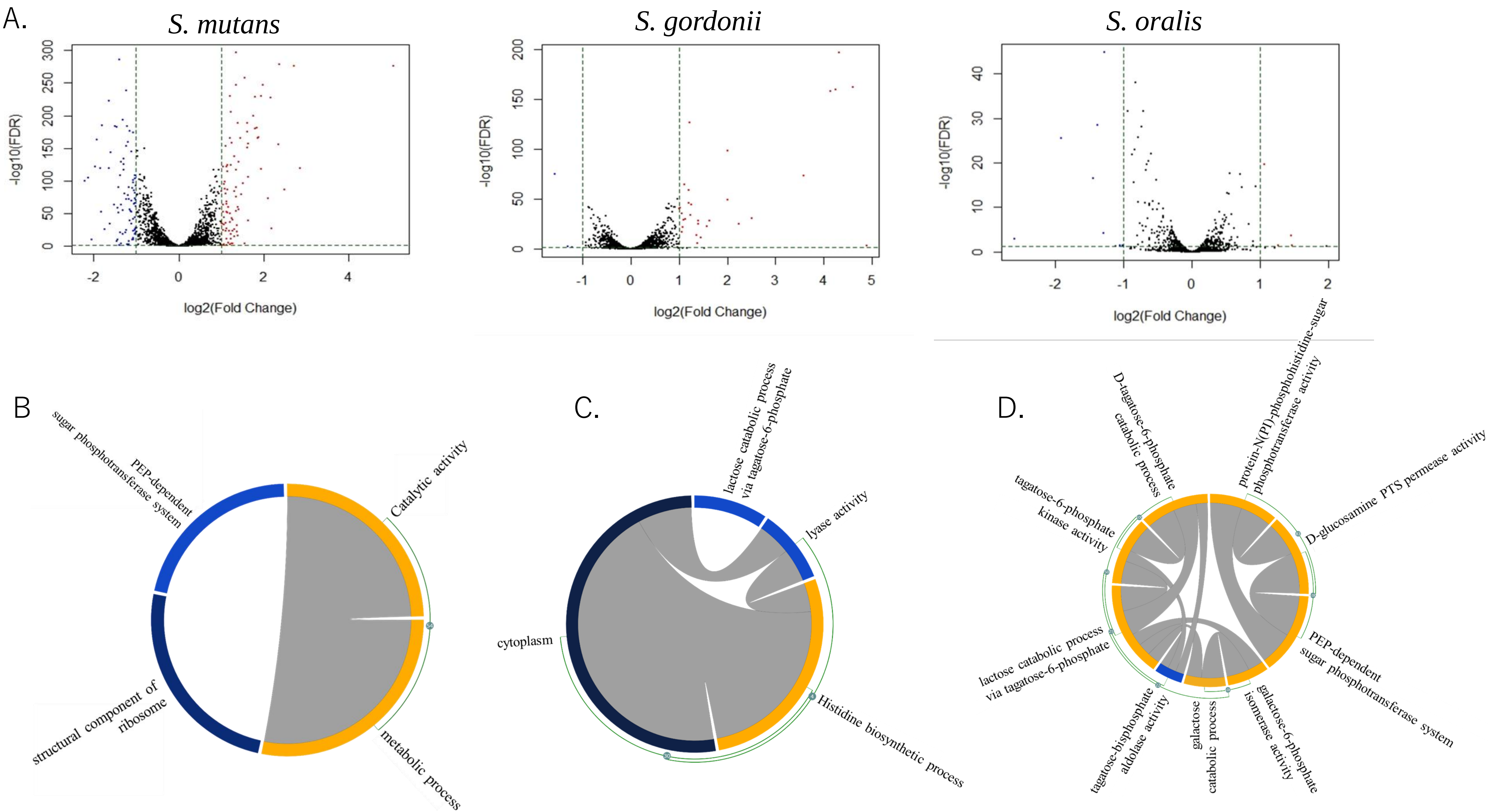

A.
S. mutans
S. gordonii
S. oralis
-log10(FDR)
log2(Fold Change)
B
sugar phosphotransferase system
PEP-dependent
Catalytic activity
structural component of ribosome
metabolic process
C.
lactose catabolic process via tagatose-6-phosphate
lyase activity
cytoplasm
Histidine biosynthetic process
D.
D-tagatose-6-phosphate catabolic process
protein-N(PI)-phosphohistidine-sugar phosphotransferase activity
D-glucosamine PTS permease activity
tagatose-6-phosphate kinase activity
PEP-dependent sugar phosphotransferase system
lactose catabolic process via tagatose-6-phosphate
tagatose-bisphosphate aldolase activity
galactose catabolic process
galactose-6-phosphate isomerase activity

E.

S. mutans *S. gordonii* *S. oralis*

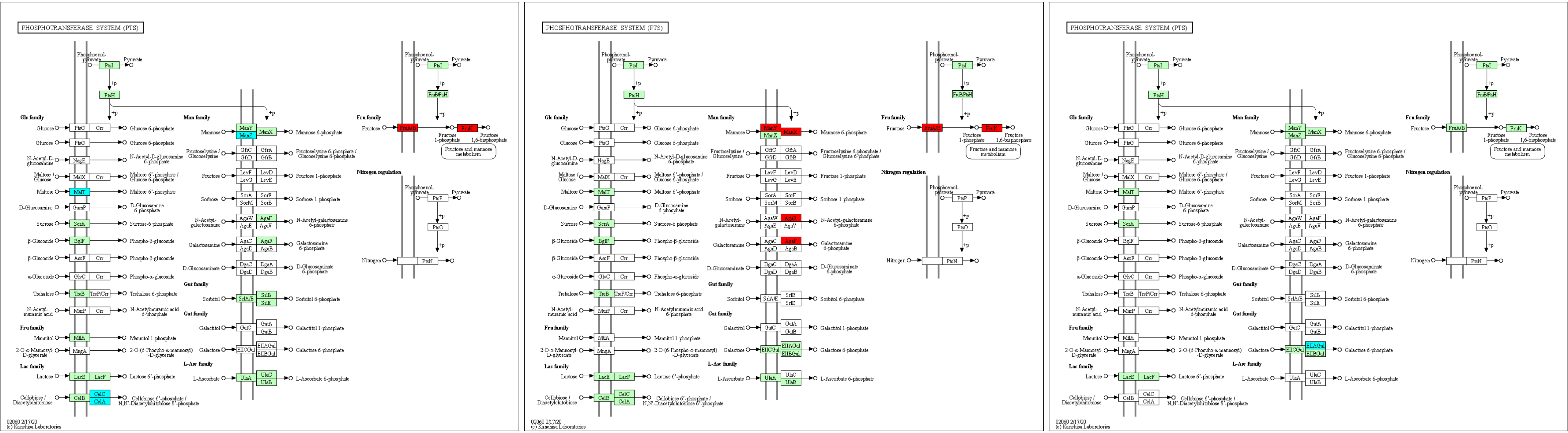

FIGURE S1. **Overview of transcriptome changes by D-tagatose.** (A) represents volcano plots of the changes in gene expression levels by D-tagatose in *S. mutans*, *S. gordonii* and *S. oralis*. In the Volcano plots, the X-axis represents $\log_2$(fold-change; FC) and the Y-axis represents $-\log_{10}$(False Discovery Rate; FDR). The vertical dotted lines indicate $\log_2 FC = \pm 1$, and the horizontal dotted line indicates FDR = 0.05. The red dots and blue dots represent differentially expressed genes with FDR < 0.05 and $|\log_2 FC| > 1$. (B), (C), and (D) show the results of GO analysis ($p < 0.05$) using Mona GO (https://monago.erc.monash.edu/). (B), (C), and (D) represent the biological processes for down-regulated DEGs in *S. mutans*, up-regulated DEGs in *S. mutans*, and up-regulated DEGs in *S. gordonii*, respectively. (E) represents the phosphotransferase system (PTS) pathways in *S. mutans*, *S. gordonii*, and *S. oralis* using KEGG Mapper (http://www.genome.jp/kegg/mapper.html). Colored boxes indicate annotated genes, red for up-regulated genes ($\log_2 FC > 1$, FDR < 0.05), blue for down-regulated genes ($\log_2 FC < -1$, FDR < 0.05).
